# Arts Flow Activities as Preventive Interventions for Stress and Anxiety in Children

**DOI:** 10.1192/j.eurpsy.2026.11857

**Published:** 2026-07-31

**Authors:** A. Argyriadi, A. Argyriadis

**Affiliations:** 1 Psychology, American University in Dubai, Dubai, United Arab Emirates; 2 Nursing, Hellenic Mediterranean University, Heraklion, Crete, Greece

## Abstract

**Introduction:**

Stress and anxiety during childhood are linked to long-term risks of psychopathology if unaddressed. School-based interventions are increasingly recognized as effective preventive strategies. Arts Flow Activities, inspired by Csikszentmihalyi’s Flow Theory, integrate creative expression and movement to foster resilience and emotional regulation.

**Objectives:**

To explore the feasibility and impact of Arts Flow Activities on stress reduction and emotional well-being in children through an in-depth case study design.

**Methods:**

A qualitative case study was conducted with a class of 25 primary school children (ages 9–10) in a community school. Over eight weeks, weekly sessions involving art, storytelling, and movement were implemented. Data collection included classroom observations, semi-structured interviews with teachers, and reflective journals from participating children. Thematic analysis was applied to identify patterns of change in emotional regulation, engagement, and stress expression.

**Results:**

Findings highlighted reduced signs of stress in classroom interactions, improved peer collaboration, and reports of positive emotional experiences among children. Teachers observed enhanced self-regulation and greater participation during academic tasks. Children described sessions as enjoyable, calming, and a source of confidence.

**Conclusions:**

The case study provides preliminary evidence that Arts Flow Activities can promote resilience and reduce stress in children. This experiential, creative approach may be integrated into preventive school-based mental health programs. Future research should expand with multiple case studies and mixed-method evaluations to assess broader applicability and outcomes.

**Disclosure of Interest:**

None Declared

